# Bulbar onset amyotrophic lateral sclerosis: A case report

**DOI:** 10.1016/j.amsu.2022.104889

**Published:** 2022-11-13

**Authors:** Prasamsa Pudasaini, Shashank Neupane, Bishal Dhakal, Abilasha Rana, Bishnu Deep Pathak, Sagun Dawadi

**Affiliations:** Nepalese Army Institute of Health Sciences, Sanobharyang, Kathmandu, Nepal

**Keywords:** Amyotrophic lateral sclerosis, Dysphagia, Fasciculation

## Abstract

**Introduction:**

Amyotrophic lateral sclerosis is a rare progressive neurodegenerative disease that affects the brain and spinal cord nerve cells. The study highlights the role of early diagnosis and prognosis of bulbar onset ALS.

**Case presentation:**

We present a case of 60 years old female who presented with slurring of speech with a deviation of tongue and progressive dysphagia. With the role of Magnetic Resonance Imaging (MRI), Electromyography (EMG) and Nerve Conduction Study (NCS), a diagnosis of ALS was made.

**Clinical discussion:**

ALS is a progressive neurodegenerative disorder, and the presence of genioglossus muscle involvement at diagnosis implies a shorter survival. It mandates multidisciplinary aspects requiring a professional opinion from neurologists, speech therapists, otolaryngologists, and electrophysiologists for effective treatment. Edaravone has antioxidant properties which counteract oxidative stress leading to neuronal injury in patients with ALS.

**Conclusion:**

ALS with bulbar onset can have a grave prognosis and hence requires a multidisciplinary approach toward effective treatment.

## Highlights

• Amyotrophic lateral sclerosis is a progressive neurodegenerative disease that affects brain and spinal cord nerve cells.
• Presence of bulbar onset can lead to poor prognosis.
• Professional opinion from multidisciplinary team is required for effective treatment.

## 1 Introduction

Amyotrophic lateral sclerosis (ALS), also known as “Lou Gehrig disease,” is a neurodegenerative disease of the motor neurons. 90–95% of cases are sporadic, the remainder being familial. The most relevant mechanism includes free radical toxicity due to superoxide dismutase type 1 mutations, defective RNA processing, excitotoxicity (excessive glutamate), and rarely due hexanucleotide repeat expansion of the C9ORF72 gene [1]. It is reported that the incidence of ALS is approximately around 1–2.6 cases per 100 000. The average age of onset of ALS is 58–60 years [2]. Advancement of disease from diagnosis to death is high with variability from 2 months to 15 years. The advancement patterns of spinal ALS are inconsistent, whereas bulbar ALS most often follows a progressive, distinguishable course [3].

Herein, we report a case of a 60-year female who was initially suspected of having a stroke based on her clinical features. However, subsequent evaluation and investigations found her suffering from ALS. The study also highlights the importance of ruling out other pathologies before diagnosing ALS and the prognosis of bulbar onset ALS. The case has been reported as per CARE 2020 guidelines [4].

## 2 Presentation of case

Sixty-year-old female, a known case of hypertension for three years, presented to our center with a history of slurring speech for three days. Slurring of speech developed suddenly, which was progressive in nature and was associated with the deviation of tongue to the left side. She also complained of difficulty swallowing, which developed insidiously, initially for solid food, which then progressed to semi-solid foods over eight months, reflecting the progressive nature of the illness. She had normal bowel and bladder habits with no history of numbness or weakness in the face, arm, or legs, and difficulty with vision or headache.

The patient's vital signs were stable with unremarkable findings on general examination. On examination of the nervous system, higher mental function, motor, and sensory systems were intact with a normal plantar response. However, the tongue was deviated towards the left side with fasciculations. Gag reflex was diminished. Baseline investigations were within normal limits. With all the presentations mentioned earlier, stroke was suspected, for which various clinical examinations and Non-Contrast Computed Tomography (NCCT) head were done. However, there was no evidence in favour of it.

Then, the patient was referred to Otolaryngologist/head and neck surgeon and speech pathologist to evaluate and manage her dysphagia and dysarthria. Therein, nasopharyngo-laryngoscopy was performed, which showed a full-length adduction gap of the vocal cord, after which speech therapy was advised. Acetylcholine receptor antibody (binding) and anti-MUSK antibody levels were <0.11 and 0.69, respectively. Nerve Conduction Velocity (NCV) showed essentially normal conduction velocity of motor and sensory nerves. Electromyography (EMG) showed fasciculations/fibrillation potentials from Genioglossus. No obvious fasciculations or fibrillation from other myotomes were noted. Intervals of EMG were suggested after three months to look for the progression of the disease. In addition, T2 weighted and FLAIR MRI images demonstrated few discrete hyperintense foci in the bilateral cerebral hemisphere's deep white matter and periventricular white matter. Further, corresponding areas showed iso-signal in T1 weighted images. Based on the clinical evaluation by neurologist and imaging findings, a diagnosis of bulbar onset amyotrophic lateral sclerosis was thus made.

Following diagnosis, injection of Edaravone 60 mg via intravenous route once daily for 14 days followed by a 14-day drug-free period in a cycle was administered as a part of treatment.

## 3 Discussion

ALS is a neurodegenerative disorder characterized by progressive muscular paralysis [5]. The foremost symptom can be either bulbar or spinal. The spinal presentation is usually stumbling or weakness of grasp [3]. Similarly, bulbar presentation is slurred or hyper nasal speech, dysphagia, hoarseness, and facial weakness. In our case, the patient had the foremost symptoms of the bulbar component, such as acute slurring of speech with a deviation of the tongue to the left side and progressive dysphagia for solid and semi-solid food.

Myasthenia gravis and multiple sclerosis usually render bulbar involvement along with ALS. The uncommon comparative occurrence of these conditions can cause delays in early diagnosis and treatment [6]. Muscle-specific kinase-positive myasthenia gravis is characterized by bulbar muscle involvement, often imitating amyotrophic lateral sclerosis with bulbar weakness [7]. In our case, myasthenia gravis was one of the differentials; however, Acetylcholine receptor antibody (binding) and anti-MUSK antibody levels were within normal limits. Nerve Conduction Velocity (NCV) and Electromyography (EMG) were performed on our patient where the latter showed fasciculations/fibrillation potentials from Genioglossus; however, no apparent fasciculations or fibrillation were traced from other myotomes. Intervals of EMG were suggested after three months to look for the progression of the disease. The latest studies suggest the involvement of the genioglossus muscle at the diagnosis implies a shorter survival, an earlier commencement of dysphagia, and severe dysarthria [3,8]. Similarly, in patients with no clinical lower motor neuron (LMN) bulbar features, the time to non-invasive ventilation was particularly associated with genioglossus abnormalities at diagnosis [8]. Bulbar onsets of symptoms can have a poor prognosis for the patient [9,10]. In our patient, EMG demonstrated fasciculations and fibrillation potentials from genioglossus sparing other myotomes. So, to look for further progression, EMG was advised after three months. MRI is generally considered a diagnostic tool to rule out alternative neurological conditions that imitate ALS. However, evaluation of the brainstem for structural, neoplastic, vascular, inflammatory, and infiltrative processes is critical [11]. MRI was also performed in our case and did not point toward such pathologies.

ALS mandates multidisciplinary aspects requiring a professional opinion from neurologists, speech therapists, otolaryngologists, and electrophysiologists for effective treatment [11]. Similarly, the multidisciplinary approach was well executed in our patient. The mainstay for managing a patient with ALS is symptomatic, as it shows promising results by increasing the survival rate and overall quality of life. The primary concern is speech therapy, the use of muscle relaxants for treating muscle stiffness, nutrition support, and respiratory care. Previously, Riluzole was considered for a patient with ALS; however, the FDA recently approved Edaravone to treat ALS [12]. Edaravone has antioxidant properties which counteract oxidative stress leading to neuronal injury in patients with ALS [13]. Likewise, our patient was treated with an Injection of Edaravone 60 mg via Intravenous route once daily for 14 days, followed by a 14-day drug-free period in a cycle. Newer modalities such as stem cell therapy are well-tolerated, but the safety and efficacy of those therapies alone or their usage in combination with other treatments are yet to be studied in detail [12].

Despite treatment, patients presenting with bulbar symptoms have shorter survival, rapid functional deterioration, and reduced quality of life. And aftermath also, ALS is still understudied and the research literature is scant [11].

## 4 Conclusion

Amyotrophic lateral sclerosis can present with predominant bulbar symptoms, leading to confusion from other neuromuscular disorders. The presence of bulbar onset of symptoms can have a grave prognosis and requires a multidisciplinary team for better treatment.

## Author agreement statement

We the undersigned declare that this manuscript is original, has not been published before and is not currently being considered for publication elsewhere.

We confirm that the manuscript has been read and approved by all named authors and that there are no other persons who satisfied the criteria for authorship but are not listed. We further confirm that the order of authors listed in the manuscript has been approved by all of us.

We understand that the Corresponding Author is the sole contact for the Editorial process. He/she is responsible for communicating with the other authors about progress, submissions of revisions and final approval of proofs.

## Ethical approval

N/A.

## Sources of funding for your research

None.

## Author contribution

Author 1: Led data collection, concept of study, contributed in writing the case information Author 2: Literature review and writing case information Author 3: Literature review, revising, writing and editing the manuscript into final version Author 4: Literature review, revising and editing the manuscript Author 5: Literature review, revising and editing the manuscript Author 6: Literature review, revising and editing the manuscript All authors were involved in manuscript drafting and revising, and approved the final version.

## Consent

Written informed consent was obtained from the patient for publication of this case report and accompanying images. A copy of the written consent is available for review by the Editor-in-Chief of this journal on request.

## Registration of research studies

1. Name of the registry: N/A.
2. Unique Identifying number or registration ID: N/A.
3. Hyperlink to your specific registration (must be publicly accessible and will be checked): N/A.

## Guarantor

Ms.Prasamsa Pudasaini, Nepalese Army Institute of Health Sciences, Sanobharyang, Kathmandu, Nepal. E-mail: prasamsapudasaini@gmail.com.

## Provenance and peer review

Not commissioned, externally peer reviewed.

## Declaration of competing interest

The authors report no conflicts of interest.
